# Access to Refugee and Migrant Mental Health Care Services during the First Six Months of the COVID-19 Pandemic: A Canadian Refugee Clinician Survey

**DOI:** 10.3390/ijerph18105266

**Published:** 2021-05-15

**Authors:** Joseph Benjamen, Vincent Girard, Shabana Jamani, Olivia Magwood, Tim Holland, Nazia Sharfuddin, Kevin Pottie

**Affiliations:** 1 Faculty of Medicine, University of Ottawa, 451 Smyth Rd, Ottawa, ON K1H 8M5, Canada; bjose093@uottawa.ca (J.B.); vgira095@uottawa.ca (V.G.); sjama085@uottawa.ca (S.J.); 2 C.T. Lamont Primary Care Research Centre, Bruyère Research Institute, 85 Primrose Ave, Ottawa, ON K1R 6M1, Canada; omagwood@bruyere.org; 3 Interdisciplinary School of Health Sciences, Faculty of Health Sciences, University of Ottawa, 75 Laurier Ave. E, Ottawa, ON K1N 6N5, Canada; 4 Department of Family Medicine, Faculty of Medicine, Dalhousie University, 1465 Brenton St, Suite 402, Halifax, NS B3J 3T4, Canada; timothy.holland@dal.ca; 5 Department of Medicine, Faculty of Medicine, University of Alberta, 11350-83 Ave, Edmonton, AB T6G 2G3, Canada; nazia.s@gmail.com; 6 Department of Family Medicine, Faculty of Medicine, University of Ottawa, 600 Peter Morand Cres, Suite 200, Ottawa, ON K1G 5Z3, Canada; 7 Institut du Savoir Montfort, 713 Montreal Rd, Ottawa, ON K1K 0T2, Canada

**Keywords:** refugees and migrants, COVID-19, mental health services, common mental health disorders, virtual care, primary care

## Abstract

The COVID-19 pandemic has had a major impact on the mental health of refugees and migrants. This study aimed to assess refugee clinician perspectives on mental health care during the COVID-19 pandemic, specifically access to and delivery of community mental health care services. We utilized a mixed methods design. We surveyed members of a national network of Canadian clinicians caring for refugees and migrants. Seventy-seven clinicians with experience caring for refugee populations, representing an 84% response rate, participated in the online survey, 11 of whom also participated in semi-structured interviews. We report three major themes: exacerbation of mental health issues and inequities in social determinants of health, and decreased access to integrated primary care and community migrant services. Clinicians reported major challenges delivering care during the first 6 months of the pandemic related to access to care and providing virtual care. Clinicians described perspectives on improving the management of refugee mental health, including increasing access to community resources and virtual care. The majority of clinicians reported that technology-assisted psychotherapy appears feasible to arrange, acceptable and may increase health equity for their refugee patients. However, major limitations of virtual care included technological barriers, communication and global mental health issues, and privacy concerns. In summary, the COVID-19 pandemic has exacerbated social and health inequities within refugee and migrant populations in Canada and challenged the way mental health care is traditionally delivered. However, the pandemic has provided new avenues for the delivery of care virtually, albeit not without additional and unique barriers.

## 1. Introduction

The COVID-19 pandemic has had a significant impact on the mental health of refugees and migrants across the globe, with greater levels of self-reported depression, worry, anxiety and loneliness among this population [1]. The mental health implications of the pandemic are particularly concerning given that common mental health disorders such as anxiety, depression and post-traumatic stress disorder are often more prevalent among refugee and migrant populations of all ages compared to the general population [2].

In Canada, refugees, migrant workers and immigrants have faced some of the highest rates of early COVID-19 positivity, despite low testing rates within this population [3]. This disparity of infections has been correlated with refugee-specific social determinants of health, such as fewer years of formal education and low English/French proficiency [3]. Public health restrictions have created unintended consequences, magnifying isolation, racial stigma, social inequities, and decreasing access to care [4]. At the same time, migrants are more likely to work in precarious front-line roles, such as taxi-cab operators, hotel housekeepers, long-term care workers, and food servers [5]. The pandemic has exacerbated unemployment, documentation problems, child and spousal abuse and relationship distress [6].

The vast majority of mental health care services for refugees and migrants are delivered in primary health care [2]. COVID-19 outbreaks locked down many clinics and dramatically reduced on-site care. This has triggered a global surge in virtual care (telemedicine), forcing patients, family physicians and clinic teams to adapt rapidly. While these virtual care tools are aimed to increase the accessibility of mental health care services, there is a paucity of data on their usage during the COVID-19 pandemic, specifically with vulnerable populations such as refugees and other migrants. Thus, the objectives of this mixed methods study are to explore general practitioner and other refugee clinician perspectives on the accessibility and delivery of mental health care, with an extra focus on virtual care, for refugees and migrants during the first 6 months of the COVID-19 pandemic. We address the following research question:

From the perspectives of clinicians who provide mental health care to refugees in Canada, how has the COVID-19 pandemic affected the mental health care needs, access to care, and management of their patients?

## 2. Materials and Methods

This study received ethical approval from the Bruyère Continuing Care Research Ethics Board (#M16-20-020), Ottawa, Ontario, Canada. We report our findings according to the Good Reporting of a Mixed Methods Study (GRAMMS) reporting guidelines [7].

### 2.1. Study Design

We conducted a sequential (quantitative→qualitative) mixed methods study consisting of a survey and follow-up interviews among clinicians who provide care to refugees across Canada. The survey contained both closed-ended and open-ended responses. We used the results of the survey to develop an interview guide, which allowed for a deeper exploration of the concepts identified by survey respondents. We triangulated quantitative and qualitative data on access barriers and opportunities during the analysis process. This mixed methods approach was selected because these types of investigations combine the generalizability offered by quantitative survey research with the deep descriptions and lived realities explored by qualitative methods—an identified strength for primary care and health services research [8,9].

### 2.2. Research Team and Reflexivity

We assembled a team of primary care providers with expertise in refugee and migrant mental health care (T.H., N.S.), qualitative research experience (O.M., K.P.), and lived experience of forced migration (Anonymous). We also included expertise in refugee mental health guidelines (K.P.) for depression, PTSD and domestic violence. The team consisted of 4 males and 3 females that represented both visible and language minorities. The team worked closely together on several COVID-19 projects.

### 2.3. Participants and Setting

Clinicians were recruited from the Canadian Refugee Health Network, a national network of primary care and specialist clinicians, including nurses and psychologists, who care for refugees and other migrants living in Canada. Eligibility criteria included provision of health care to recently arrived refugee patients during the COVID-19 pandemic. We used a series of three emails to enroll network members in the online survey. We used snowball sampling to enlist participants for the qualitative interviews, with network members nominating specific colleagues for invitations to participate. We continued recruitment until theoretical saturation was achieved, defined as when subsequent interviews contribute no new concepts [10]. We estimated that this would occur at 10–13 interviews [11].

### 2.4. Questionnaire

A bilingual (English–French) survey questionnaire was developed with closed and open-ended questions (see Supplementary File S1: Survey). One family physician (K.P.) and one medical student (J.B.) adapted several of the survey questions from a recent international survey conducted among refugee clinicians in Canada, the US, Australia and Ireland [12]. Specifically, we adapted categorical questions on provider characteristics (profession, gender, years of experience with refugee care), patient characteristics (languages spoken, country of origin), and interpreter availability. The English survey was initially translated by a francophone member of our study team (V.G). This translation was further verified by other French-speaking team members (O.M., K.P). Survey participants were provided with the option to complete the survey in either English or French. None of the participants indicated that they would like to complete the survey in French. The survey was pilot tested on three experienced clinicians (T.H., N.S., M.W.) and revised for conciseness and clarity. The online survey collected information on mental health interventions offered to refugees, challenges faced by clinicians in offering care, and what alternative services clinicians believe would allow them to optimize the delivery of care during the COVID-19 pandemic.

A bilingual (English–French) follow-up semi-structured interview guide was developed to further explore and contextualize survey responses (see Supplementary File S2: Interview Guide). The interview guide consisted of five key questions and additional prompts. These questions asked about the mental health status of refugee patients, mental health care access and management strategies at the clinician practice, both current and projected. Bilingual team members independently verified the French translation (V.G., K.P., O.M.).

### 2.5. Data Collection

The survey was available to clinicians online for a period of 75 days between May and July 2020. Semi-structured interviews were conducted between July and August 2020. Interviews were conducted until data saturation was achieved. To respect public health social distancing recommendations during the COVID-19 pandemic, interviews were conducted over the phone or using secured, encrypted video conferencing software by two medical students (J.B. and V.G.). Interviews were scheduled for 60 min and were audio-recorded. We reflected statements back to participants during the interview as a form of immediate member checking. We transcribed the recordings verbatim using the transcription software Otter and Sonix and we reviewed all transcripts for accuracy [13,14].

### 2.6. Analysis

Participant demographics were summarized using descriptive statistics. Responses to closed-ended survey questions were described using frequencies and percentages. Responses to open-ended survey questions and interviews were analyzed inductively using the framework method [15]. Three research team members (J.B., S.J., V.G.) familiarized themselves with the transcripts and began independently open-coding a subset of transcripts to identify emerging themes. The research team then discussed and agreed upon a set of codes to apply to subsequent transcripts. An analytical framework consisting of 3 themes, 8 categories and 40 codes was developed. This analytic framework was applied independently, in duplicate, to all transcripts. Disagreements in indexing of transcripts were resolved through discussion. Interpretation was performed through iterative discussions with the entire research team. We triangulated our quantitative and qualitative data. Key qualitative findings were juxtaposed with closed-ended survey responses and reported according to major themes. As a final member checking exercise, we asked key clinician experts to comment on our draft findings.

## 3. Results

### 3.1. Description of Participants

Ninety-two general practitioners and other primary care clinicians self-identified as eligible for our survey and completed study registration. Of these, 77 clinicians, all of whom provide mental health services to refugees and migrants, completed the survey, representing a response rate of 84%. The online survey took an average of 14 min to complete. Table 1 presents survey participant characteristics. Eleven of these clinicians participated in qualitative interviews to explore access to care, mental health care and virtual mental health care during COVID-19.

### 3.2. Main Themes

Results from the survey and interviews provide an overview of clinicians’ understanding of the impact of the pandemic on their refugee and migrant patients, challenges with managing refugee mental health and perspectives on improving care, as well as impressions of virtual care.

### 3.3. Effects of the Pandemic on Refugee Health

Clinician participants reported that the COVID-19 pandemic has affected the health of their refugee patients in three major ways: negative mental health condition outcomes, exacerbation of migrants’ social determinants of health and decreased access to services and community resources.

#### 3.3.1. Negative Mental Health Outcomes

Negative mental health outcomes included increased incidence and exacerbations of mental health conditions, increased demand for mental health services and themes related to uncertainty, fear, worry, and stress. More than half of the clinicians surveyed (52%) reported an increase in refugee patients requesting anxiety or mental health support (Table 2).

*“The city we are in looks like it is under siege and some have commented that it appears as a war struck area, instigating their PTSD.”*

—Urban Family Physician with over 15 years of experience caring for refugees and migrants.

*“Most of my clients disclosed that the pandemic triggers them, because they have to stay inside, and this reminded them of the war time, as they should hide from the conflicts and the death outside their doors.”*

—Clinical Counsellor with less than 5 years of experience caring for refugees and migrants.

#### 3.3.2. Exacerbation of Social Determinants of Health

In addition to the negative mental health impact of the pandemic, exacerbation of social and economic conditions was also a common theme. In particular, income and financial issues, disruption of family dynamics, isolation and housing issues were commonly noted social determinants of mental health. Regarding issues in income, more than half of responding clinicians (53%) had the impression that there was an increased rate of unemployment among their refugee patients during the COVID-19 pandemic (Table 3).

*“Scared, unsure, confused, worried. Families are living in cramped quarters. Children have nowhere to go. Food at times was short. Access to any type of health care curtailed. Poverty is exemplified in such a situation.”*

—Suburban Nurse Practitioner with 10–15 years of experience caring for refugees and migrants.

#### 3.3.3. Decreased Access to Health Care Services

A major theme that emerged within the impact of the pandemic on refugees was decreased access to health care services and decreased access to community resources. Among the 77 clinicians surveyed, 52 (68.42%) noted that refugee access to care was lower during the pandemic (Table 3). In addition to reduced clinic visits, decreased access to social supports, including child care, language classes, and resettlement services, was a frequently noted sub-theme. Furthermore, fear of exposure and lack of information on available services was commonly reported as a reason for patients not seeking care.

### 3.4. Challenges with Managing Refugee and Migrant Mental Health Conditions

**Figure 1:**
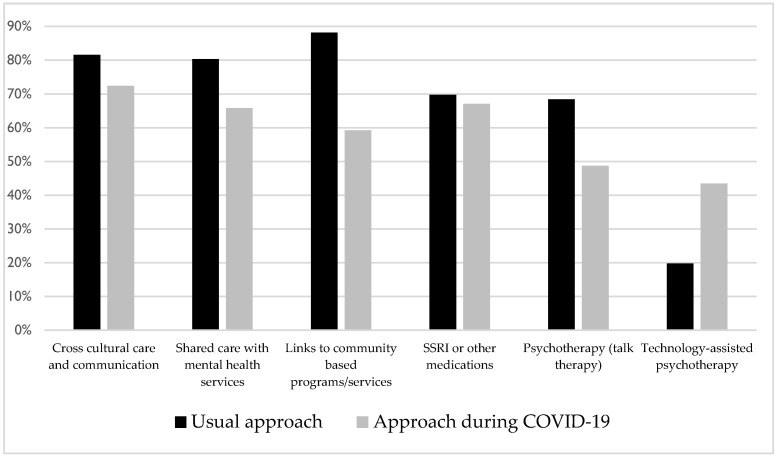
Clinician management approaches for refugee and migrant mental health before and during the COVID-19 pandemic (*n* = 76).

Twenty-nine percent fewer participants reported the use of links to community-based settlement programs and services during COVID-19 compared to their usual management practices (88.16% vs. 59.21%) (Figure 1). This was coupled with a 24% increase in participants reporting the use of virtual care psychotherapy (19.74% vs. 43.43%) and a 20% decrease in the use of psychotherapy (68.42% vs. 48.68%) (Figure 1). The two most frequently noted challenges with managing refugee mental health during COVID-19 included access to health care service issues and challenges with virtual care.

#### 3.4.1. Access to Mental Health Care Services

Within the theme of access to care, limited availability of providers and community resources, technological barriers and patients not seeking care due to reasons such as fear of exposure or perceived lack of services were the most commonly noted challenges clinicians experienced in their management.

*“The already limited available services for counselling support became even more unavailable during the pandemic, exactly at the time when we needed them more.”*

—Small-city Family Physician with 10–15 years of experience caring for refugees and migrants.

*“Due to fear many of the services that help these people are closed. Due to fear we left the most vulnerable exposed and alone. Never mind delivery of mental healthcare we barely delivered any care at all.”*

—Nurse Practitioner with 10–15 years of experience caring for refugees and migrants.

#### 3.4.2. Virtual Primary Care

The most common reported limitation of providing primary care virtually included technological barriers, such as access to technology and technological literacy. Communication issues, including challenges connecting to patients emotionally, interpreter issues, as well as privacy concerns with conducting sessions at home were also commonly noted limitations. Furthermore, difficulties with assessing patients and providing full scope of care were also noted limitations with virtual care.

*“Given there is often also a language barrier, they have more difficulty in calling the office and getting complex instructions on how to set up virtual care on their end. If they are successful at all, it will frequently end up being a phone call appointment as opposed to a video appointment, and I feel with this particular population a lot can be missed if we lack the visual cues that I often rely on to better understand my patient and make myself understood as well.”*

—Family Physician with 5–10 years of experience caring for refugees and migrants.

Despite the noted limitations, there were several reported benefits with providing virtual primary care. Increasing access to care was a major theme that emerged within the benefits of virtual care. In particular, emerging sub-themes included addressing transportation and childcare issues, decreased wait-times and the ability to connect after-hours and to more providers. Other benefits of virtual care included increased comfort for patients and the ability to visualize a patient’s living context. Approximately 65% of respondents reported that technology-assisted psychotherapy is feasible to arrange and acceptable for the primary care of refugees (Table 4). Furthermore, approximately 67% of clinician participants believe that technology-assisted psychotherapy could increase health equity for their refugee patients (Table 4).

### 3.5. Improving Care for Refugees and Migrants

Clinicians’ perspectives on improving refugee and migrant mental health care during the pandemic and beyond were also assessed. The two most frequently noted themes included increasing access to community resources and virtual care. In particular, clinicians reported that increasing access to counselling, interpretative services, social supports, and navigation or outreach services would improve mental health care for their patients. Within the theme of access to virtual care, increasing access to technology, addressing technological illiteracy, and increasing funding for virtual care were frequently noted sub-themes. Additional themes related to advocacy also emerged, including advocating for structural and organizational change, addressing social determinants of health and increasing funding for refugee health care.

## 4. Discussion

This study shows that in the first 6 months of the pandemic, COVID-19 was insidiously harming refugees and migrants [3]. Clinicians working with refugees reported a pan-Canadian use of virtual care to support refugee mental health conditions. Clinicians also emphasized the importance of social determinants of health and access to primary health care to support mental health. COVID-19 and the unique social and medical management needed for refugee care amplified clinician challenges and we discuss these issues.

Social determinants of health and health systems play a critical factor in determining refugee and migrant patient access to primary health care [16]. Indeed, prior to COVID-19, refugee access to care was limited due to income, unemployment, literacy, housing, social exclusion, race and official language skills [17]. Our results suggest an exacerbation of barriers and of mental health conditions related to the COVID-19 pandemic. For example, many clinicians reported that the newly established jobs of their refugee patients disappeared as businesses shut down. Language and integration settlement classes were cancelled, leading to further social isolation and maintaining education disparities between refugees and migrants and Canadian-born individuals. Many clinicians reported that their refugee patients were barely able to afford satisfactory housing and faced evictions due to loss of income, despite Canadian government attempts to prevent evictions during the pandemic restrictions. The recent WHO Apart Together Survey also shows the stigma and mental health impacts of the COVID-19 pandemic on migrants around the world [1].

Clinicians in our survey reported that refugees could not access the basic health or mental health services they required. Data from other host countries report that refugees are 60% more likely than the host population to work in sectors most impacted by the pandemic, such as accommodation and food services, meat processing, long-term care and retail [18,19]. COVID-19 may create a “perfect storm” for certain refugees who relive their previous war-time traumas in isolation due to fears and pandemic restrictions. In addition to incurring daily stresses, refugees and migrants are trying to restart their lives in a new country but now with limited access to education or job opportunities. Prior experiences and trust within their communities may support refugees in surviving these restrictions and losses [20]. Clinicians reported that many of their refugee patients drew strength from their experiences living through infectious disease outbreaks in refugee camps. Building on these experiential strengths may require cultural and global mental health skills and supports [21].

Virtual care may offer opportunities in refugee mental health care, eliminating transportation costs, improving triage and timely access, and linking to other virtual resources [22]. Early research suggests refugees can benefit from virtual care; however, more equity and technology access research is needed for cultural and linguistically diverse populations [23]. For example, clinicians reported that certain refugees expressed a desire for on-site visits with their trusted clinician to reduce angst and anxiety related to COVID-19. As well, many refugees could not afford computers, tablets or internet access and did not have the language skills and computer literacy that were needed to access virtual care.

Strengths: This national mixed methods study was conducted in Canada’s two official languages, English and French. The study took place in the first six months of the COVID-19 pandemic and focused on real world practitioners with experience caring for recently arriving refugees and migrants. The inclusion of semi-structured qualitative interviews provided opportunities to examine the personal effect of COVID-19 on clinicians.

Limitations: The study surveyed and interviewed refugee health clinicians, not refugee patients. More than 50% of our participants were from Ontario, the same province that receives more than 50% of refugees to Canada. A sensitivity analysis showed that Ontario is the most culturally and linguistically diverse province in Canada, has a significant Franco-Ontario population, and has special initiatives to resettle refugees in small and mid-size cities [24]. However, it is possible that Ontario refugee clinics may be more developed than other areas of the country. Participants self-identified and volunteered as eligible, and thus, this sample was not necessarily representative of all clinicians in Canada. Refugee and migrant access, isolation, and socioeconomic challenges may be context-dependent and dynamic throughout the pandemic. Virtual care is emerging as a powerful tool in primary health care, but we need to measure the equity of access, experiences, and effectiveness more precisely in front-line refugee and migrant care.

## 5. Conclusions

The COVID-19 pandemic has struck refugees and migrants with infections and social isolation, impairing access to care, increasing social inequity and revealing shortcomings with virtual care. Examples include loss of jobs and income, accenting stigma and structural racism and reducing community organization support. Virtual approaches to care have emerged as a promising new delivery tool that may eventually reduce barriers and cost to care but may demand strong global mental health skills. However, more research is needed to ensure equity of access to virtual services. This study of clinicians working with refugees across Canada shows the importance of advocacy and innovation in the context of social equity and trust during the COVID-19 pandemic. Access to primary mental health care remains vulnerable and may benefit from virtual care approaches; however, further research on effectiveness and equitable access is needed.

## Figures and Tables

**Table 1:** Survey participant (clinician) demographic information.

Characteristic	*N*	%
**Profession**		
Physician * Family Physician (29), Psychiatrist (9),* * Pediatrician (5), Emergency Physician (3),* * Internist (3)*	49	63.64
Nurse or Nurse Practitioner	12	15.58
Psychologist or Counselor	12	15.58
Other * Social Worker (2), Program Lead (1),* * Client Advocate (1)*	4	5.19
**Gender**		
Female	56	72.73
Male	20	25.97
Prefer not to say	1	1.30
**Length of Time Caring for Refugees**		
<5 years	22	28.57
5–10 years	25	32.47
10–15 years	10	12.99
15+ years	20	25.97
**Province or Territory**		
Alberta	11	14.29
British Columbia	11	14.29
Newfoundland and Labrador	1	1.30
Nova Scotia	7	9.09
Ontario	42	54.55
Prince Edward Island	1	1.30
Quebec	3	3.90
Saskatchewan	3	3.90
Nunavut	1	1.30
Yukon	1	1.30
**Received Training in Refugee Mental Health Care Approaches**		
Yes	48	62.34
No	29	37.66
**Psychologists or Psychiatrists Working Within or Linked to Clinic**		
Yes	51	67.11
No	25	32.89
**Availability of Medical Interpretation Services Within Clinic**		
Yes	66	85.71
No	10	12.99
Don’t Know	1	1.30

**Table 2:** Clinician perspectives on the effects of the pandemic on the mental health of refugees and other migrants.

Survey Question	*N*	Yes *n* (%)	No *n* (%)	Don’t Know *n* (%)
Has the pandemic resulted in an increase in refugee and other migrant patients requesting anxiety or mental health support?	77	40 (51.95)	21 (27.27)	16 (20.78)
During COVID-19 have you noted increased tension and conflict within refugee family relationships?	77	37 (48.05)	20 (25.97)	20 (25.97)

**Table 3:** Clinician perspectives on the effects of the pandemic on refugee and other migrant social determinants of health.

Survey Question	*N*	Higher *n* (%)	Similar *n* (%)	Lower *n* (%)	Don’t Know *n* (%)
Do you have the impression that your refugee and other migrant patients have a higher, lower or similar rate of unemployment during COVID-19 compared to pre-COVID-19?	77	41 (53.25)	10 (12.99)	12 (15.58)	14 (18.18)
Do you have the impression that refugees and other migrants have a higher, lower or similar rate of homelessness or precarious housing during COVID-19 compared to pre-COVID-19?	77	21 (27.27)	36 (46.75)	6 (7.79)	14 (18.18)
Do you have the impression that refugees and other migrants in your practice have a higher, lower or similar rate of access to care during COVID-19 compared to pre-COVID-19?	76	4 (5.26)	17 (22.37)	52 (68.42)	3 (3.95)

**Table 4:** Clinician perspectives on virtual mental health care for refugee and other migrant patients.

Survey Question	*N*	Yes *n* (%)	No *n* (%)	Don’t Know *n* (%)
Is technology-assisted psychotherapy feasible to arrange for refugees and other migrants?	75	49 (65.33)	12 (16.00)	14 (18.67)
Is technology-assisted psychotherapy acceptable for refugees and other migrants?	74	48 (64.86)	7 (9.46)	19 (25.68)
Would technology-assisted psychotherapy increase health equity for refugees and other migrants?	75	50 (66.67)	9 (12.00)	16 (21.33)

## Data Availability

The data presented in this study are available on request from the corresponding author. The data are not publicly available due to privacy restrictions.

## Supplementary Materials

The following are available online at https://www.mdpi.com/article/10.3390/ijerph18105266/s1, File S1: Survey; File S2: Interview Guide.
