# Supplementary material for: Access to Refugee and Migrant Mental Health Care Services during the First Six Months of the COVID-19 Pandemic: A Canadian Refugee Clinician Survey
Source: Int J Environ Res Public Health. 2021 May 15;18(10):5266. doi: 10.3390/ijerph18105266 (PMC8156129; doi:10.3390/ijerph18105266)
Supplement: Supplementary file 1 [file ijerph-18-05266-s001.zip › File S1- Survey.pdf]

Refugee Mental Healthcare in a Time of COVID-19

Part 1/4: Clinician Participant Demographics

* 2. Please enter your e-mail address for administrative purposes:

Email Address

3. What is your age?

- ○ Less than 40 years
- ○ 40 to 59 years
- ○ 60 years or older
- ○ Prefer not to say

4. What is your gender?

- ○ Male
- ○ Female
- ○ Non-binary or transgender
- ○ Prefer not to say

5. Do you identify as a visible minority?

- ○ Yes
- ○ No
- ○ Prefer not to say

6. What is your profession?

- ○ Physician
- ○ Nurse or Nurse Practitioner
- ○ Psychologist or Counselor
- ○ Public Health Practitioner
- ○ Other (please specify)

7. In what province(s) or territory do you work?

- ☐ Alberta
- ☐ British Columbia
- ☐ Manitoba
- ☐ New Brunswick
- ☐ Newfoundland and Labrador
- ☐ Nova Scotia
- ☐ Ontario
- ☐ Prince Edward Island
- ☐ Quebec
- ☐ Saskatchewan
- ☐ Nunavut
- ☐ Northwest Territory
- ☐ Yukon

8. In what postal code is your practice located?

9. What type of community practice is your clinic?

- ○ Special service/clinic for refugees
- ○ Special service/clinic for immigrants
- ○ Generalist primary care service/clinic
- ○ Other, please specify (e.g, Community Health Centre, Family Health Team, Fee for Service, Hospital Clinic)

10. How many years of experience of care with refugees and other migrants in Canada and related work with refugees internationally do you have?

- ◯ <5 years
- ◯ 5-10 years
- ◯ 10-15 years
- ◯ >15 years
- ◯ Prefer not to say

11. Have you ever received training in any refugee mental health care approaches?

- ◯ Yes
- ◯ No

If yes, please list which ones (i.e. psychotherapy, medication management of common mental health disorders, collaborative community approaches)

12. Do you have psychologists or psychiatrists working within or linked to your clinic setting?

- ◯ Yes
- ◯ No

13. What languages do "*you"* speak fluently with your patients?

14. Is there a medical interpretation service available at your practice?

- ◯ Yes
- ◯ No
- ◯ Don't know

INSTITUT DE RECHERCHE
BRUYÈRE
RESEARCH INSTITUTE

uOttawa

Refugee Mental Healthcare in a Time of COVID-19

Part 2/4: Effects of COVID-19 on Refugee Patients

15. Do you have the impression that your refugee and other migrant patients have a higher, lower or similar rate of unemployment during COVID-19 compared to pre-COVID-19?

- ◯ Higher
- ◯ Lower
- ◯ Similar
- ◯ Don't know

Comments

16. Do you have the impression that refugees and other migrants have a higher, lower or similar rate of homelessness or precarious housing during COVID-19 compared to pre-COVID-19?

- ◯ Higher
- ◯ Lower
- ◯ Similar
- ◯ Don't know

Comments

17. Do you have the impression that refugees and other migrants in your practice have a higher, lower or similar rate of access to care during COVID-19 compared to pre-COVID-19?

- ◯ Higher
- ◯ Lower
- ◯ Similar
- ◯ Don't know

Comments

18. Has the pandemic resulted in an increase in refugee and other migrant patients requesting anxiety or mental health support?

- ◯ Yes
- ◯ No
- ◯ Don't know

Comments

19. Have you noted refugee traditional beliefs to be helpful or harmful in coping with COVID- 19?

- ◯ Helpful
- ◯ Harmful
- ◯ Don't know

Comments

20. During COVID-19 have you noted increased tension and conflict within refugee family relationships?

- ◯ Yes
- ◯ No
- ◯ Don't know

Comments

21. Overall, how you describe the effect of the pandemic on your refugee or migrant patients? (e.g. effect on mental and physical health, socioeconomic factors, family dynamics, etc.)

INSTITUT DE RECHERCHE
BRUYÈRE
RESEARCH INSTITUTE

uOttawa

Refugee Mental Healthcare in a Time of COVID-19

Part 3/4: Practice Characteristics

22. Are you comfortable diagnosing common mental health conditions (e.g. anxiety, depression or PTSD) in refugees (aged 15 years and older)?

- ○ Yes
- ○ No
- ○ Prefer not to say

What conditions are you uncomfortable identifying?

23. What type of approach or service do you usually use in managing the mental health of your refugee/migrant patients? (Check all that apply)

- ☐ Cross cultural care and communication
- ☐ Shared care with mental health services
- ☐ Links to community based programs/services
- ☐ Other (please specify)
- ☐ SSRI or other medications
- ☐ Psychotherapy (talk therapy)
- ☐ Technology-assisted psychotherapy

24. What type of approach or service are you currently using to manage the mental health of your refugee/migrant patients **during COVID-19**? (Check all that apply)

- [ ] Cross cultural care and communication
- [ ] SSRI or other medications
- [ ] Shared care with mental health services
- [ ] Psychotherapy (talk therapy)
- [ ] Links to community based programs/services
- [ ] Technology-assisted psychotherapy
- [ ] Other (please specify)

25. Have you ever referred your refugee or migrant patients for technology-assisted psychotherapy?

- ( ) Yes
- ( ) No
- ( ) Don't know

If no, why not?

26. Do you feel that technology-assisted psychotherapy is feasible to arrange for the primary care of refugees and other migrants?

- ( ) Yes
- ( ) No
- ( ) Don't know

Please explain

27. Do you feel that technology-assisted psychotherapy would be acceptable for refugee or migrant patients?

- ( ) Yes
- ( ) No
- ( ) Don't know

List any positive or negative impacts

28. Do you believe that technology-assisted psychotherapy would increase health equity for your refugee and other migrant patients?

- Yes
- No
- Don't know

Comments

29. What concerns you the most about your ability to provide mental healthcare to refugee and other migrant patients during the pandemic?

30. In your opinion, what additional services would best support refugee and other migrant patients requiring mental health support during the pandemic?

INSTITUT DE RECHERCHE BRUYÈRE RESEARCH INSTITUTE | uOttawa

Refugee Mental Healthcare in a Time of COVID-19

Part 4/4: Refugee and Migrant Patients Treated

31. What is the region of origin of your refugee patients (select one or more)?

- ☐ North/sub-Saharan Africa
- ☐ Central and Southern Africa
- ☐ Middle East
- ☐ Eastern and Central Europe
- ☐ Indian subcontinent/South Asia
- ☐ South East Asia
- ☐ East/North Asia
- ☐ Oceania/Pacific
- ☐ Other (please specify)

32. To the best of your knowledge, which factors or barriers are “most” important in limiting access to your clinic during the pandemic?

- ☐ Language barriers
- ☐ Transportation barriers
- ☐ Family and community demands
- ☐ Low health literacy
- ☐ Full or partial clinic closure due to COVID-19

Other/Please provide an example

INSTITUT DE RECHERCHE
BRUYÈRE
RESEARCH INSTITUTE

uOttawa

Refugee Mental Healthcare in a Time of COVID-19

Conclusion

33. Please write any other information you would like to provide.

34. Please provide the names and email addresses of any clinical or mental health clinician colleagues whom you believe would be interested in participating in this study.

35. Would you be interested in participating in a linked qualitative interview to describe your experience with managing refugee mental health during the COVID-19 pandemic?

- ○ Yes
- ○ No

Survey Feedback

Thank you for completing this survey. We would appreciate your feedback on the survey:

36. Time it took you to complete (too long, just right)

37. Were the questions understandable? Any that you found confusing?

38. Were there any questions missing—things you wished we had asked?
