# Supplementary material for: Access to Refugee and Migrant Mental Health Care Services during the First Six Months of the COVID-19 Pandemic: A Canadian Refugee Clinician Survey
Source: Int J Environ Res Public Health. 2021 May 15;18(10):5266. doi: 10.3390/ijerph18105266 (PMC8156129; doi:10.3390/ijerph18105266)
Supplement: Supplementary file 1 [file ijerph-18-05266-s001.zip › File S2- Interview Guide.pdf]

Refugee Mental Healthcare in a Time of COVID-19: Interview Guide

<u>Introduction Script:</u>

Thank you for agreeing to speak with me today about the management of refugee mental health during the COVID-19 pandemic. The interview should take no longer than 60 minutes. The information you share with me today will be transcribed and de-identified. No one outside the research team will be able to identify you from the results of this study. There is no right or wrong answer to these questions, I am interested in your opinions. If you do not wish to answer a question, please let me know. You can decline to answer any questions without providing a reason. If you have any questions, please let me know. Before I begin, do I have your permission to record this interview? Do you have any questions before we begin? (START RECORDING) This is interview (ID#) on (DATE) and he/she/they understand that this will be recorded, is this correct?

1. What has your experience been like managing the mental health of your refugee patients during COVID-19?
    - How are you providing mental healthcare to your refugee patients during the pandemic?
    - How has your management of refugee mental health changed?

2. What changes to healthcare delivery do you believe would best support the mental health of your refugee patients during the pandemic and beyond?

3. What are your thoughts on virtual mental health care approaches for refugees?
    - Are they acceptable/useful to you and your refugee patients?
    - Do you see virtual mental health approaches useful for refugees in the future?

4. The results from the survey highlighted significant access to care concerns. How do you foresee this will affect the future mental health needs and management of your refugee patients?

5. Would you like to share anything else about your experience in managing the mental health of your refugee patients during the pandemic that we have not discussed today or through the survey?
